# Procedural application of mode-of-action and human relevance analysis: styrene-induced lung tumors in mice

**DOI:** 10.1080/10408444.2024.2310600

**Published:** 2024-03-05

**Authors:** Evan A. Frank, M.E. (Bette) Meek

**Affiliations:** a National Institute for Occupational Safety and Health, Cincinnati, OH, USA; b School of Epidemiology and Public Health in the Faculty of Medicine, University of Ottawa, Ottawa, Canada

**Keywords:** Human relevance of animal carcinogens, carcinogenic mode of action, human relevance framework, weight of evidence analysis, styrene

## Abstract

Risk assessment of human health hazards has traditionally relied on experiments that use animal models. Although exposure studies in rats and mice are a major basis for determining risk in many cases, observations made in animals do not always reflect health hazards in humans due to differences in biology. In this critical review, we use the mode-of-action (MOA) human relevance framework to assess the likelihood that bronchiolar lung tumors observed in mice chronically exposed to styrene represent a plausible tumor risk in humans. Using available datasets, we analyze the weight-of-evidence 1) that styrene-induced tumors in mice occur through a MOA based on metabolism of styrene by Cyp2F2; and 2) whether the hypothesized key event relationships are likely to occur in humans. This assessment describes how the five modified Hill causality considerations support that a Cyp2F2-dependent MOA causing lung tumors is active in mice, but only results in tumorigenicity in susceptible strains. Comparison of the key event relationships assessed in the mouse was compared to an analogous MOA hypothesis staged in the human lung. While some biological concordance was recognized between key events in mice and humans, the MOA as hypothesized in the mouse appears unlikely in humans due to quantitative differences in the metabolic capacity of the airways and qualitative uncertainties in the toxicological and prognostic concordance of pre-neoplastic and neoplastic lesions arising in either species. This analysis serves as a rigorous demonstration of the framework’s utility in increasing transparency and consistency in evidence-based assessment of MOA hypotheses in toxicological models and determining relevance to human health.

## Introduction

Chronic exposure to styrene causes increase in adenomas and adenocarcinomas of the bronchiolar and bronchioloalveolar regions in some strains of mice. This was first reported in male B6C3F1 mice exposed to styrene *via* gavage for 78weeks with a 13-week observation period (NCI 1979). Cruzan et al. (2001) demonstrated these tumor increases in male and female CD-1 mice exposed to inhaled styrene in a two-year cancer bioassay. Bronchiolar tumors were not observed in male or female F344 or CD (Sprague-Dawley) rats exposed to styrene by oral or inhalation routes (NCI 1979; Beliles et al. 1985; Cruzan et al. 1998). In addition, not all mouse strains develop an increase in bronchiolar tumors after styrene exposure. In a comparison study, CD-1 mice developed the expected tumors whereas C57Bl/6 mice did not, despite being exposed to up to 120 ppm inhaled styrene for the full 2-year period (Cruzan et al. 2017). These reports have prompted discussion of the human relevance of styrene-induced bronchiolar tumors.

In the period since the initial publication of data demonstrating different susceptibilities in rats and mice, discussion of possible human relevance has focused on Cyp2F2, an enzyme present in mouse bronchiolar club cells but not in rats or humans. Metabolism of styrene by Cyp2F2 has been hypothesized as an obligatory key event in the development of bronchiolar tumors by multiple authors (Cruzan et al. 2018; Hill and Conolly 2019). If this hypothesis is correct, then it follows that these tumors would not be relevant in an assessment of human health risks. The objective of this assessment is to review available data and evaluate the weight-of-evidence (WOE) that styrene-induced bronchiolar tumors in mice arise *via* a Cyp2F2-dependent MOA and the likelihood that such an MOA could occur in humans.

We utilize the WOE framework by Meek, Palermo, et al. (2014) developed specifically to assess whether evidence supports the MOA hypothesis. The framework is based on Bradford Hill causality considerations streamlined and tailored to best accommodate data informing biological MOA. This allows for a transparent assessment determining an overall WOE for different MOA hypotheses that may then be used to judge human relevance based on whether the key events/key event relationships within that MOA also are known to occur in humans. The essential questions to be answered are: 1) Does the evidence and biological context support the MOA in animal data, and 2) If it is possible to establish that the MOA occurs in animals, is it plausible that a similar MOA occurs in humans?

Five evolved Bradford Hill causality considerations are evaluated to determine whether evidence supports a given MOA hypothesis based on animal data. These are:

- Biological Concordance: Broader knowledge about the structural and functional relationships represented by the key event relationships (KERs) supports the MOA hypothesis when the proposed functional underpinnings are consistent with known biology. The impact of this on the level of confidence in the MOA should be based on overall concordance of the KER sequence as a whole. Uncertainty in understanding how specific KERs would occur functionally should be considered in context with other considerations and does not necessarily rule against the plausibility of the MOA hypothesis.
- Essentiality of Key Events: There should be a predictable impact on subsequent key events and the outcome of interest if earlier key events are modified in a way that specifically affects their functioning. This could mean that if a gene for a key protein is genetically knocked out or reintroduced, or an agent that inhibits a key component is applied, then subsequent key events and/or the outcome of interest is abolished or restored accordingly.
- Concordance of Empirical Observation with Key Events: Available experimental data should be concordant with the hypothesized arrangement of key events in terms of dose–response, temporal order, and incidence. With respect to dose-response, early key events should be observed at similar or lower doses than later key events. With respect to temporality, early key events should be observed earlier than later ones. With respect to incidence, early key events should occur at a greater frequency than later ones given the same dose or level of severity in a sample group.
- Consistency: Consistency is demonstrated by testing similar stimuli in different test systems. If the toxicant or stimulus is tested in a variety of animal strains, animal species, or other testing models, observations from those systems should be logically consistent with both the proposed MOA and the known differences and/or similarities between those test systems.
- Analogy: Analogy is demonstrated by testing different stimuli in similar test systems. If other toxicants or stimuli share the same toxic moiety or molecular initiating feature as the chemical of interest, observations from comparable test systems should be expected to bear similarity to each other as consistent with the MOA understanding.

The premise of the proposed MOA is that metabolism of styrene by Cyp2F2 causes toxicity to bronchiolar epithelial cells by generating one or more species of toxic metabolites. The exact mechanism(s) mediating toxicity through toxic metabolites are not known. A suite of studies in multiple strains of mice demonstrate initial toxicity consisting of cell death and necrosis and increases in bronchiolar cell proliferation (Cruzan et al. 1997; Green et al. 2001; Andersen et al. 2017, 2018). These studies demonstrate that with repeated exposures, the initial toxicity resolves into focal crowding of cells in the first several weeks of exposure. Focal bronchiolar hyperplasia becomes evident after several months and continues to develop throughout the full two-year cancer bioassay exposure duration. It is hypothesized that the hyperplastic response drives the increase in bronchiolar tumors seen at 104 weeks.

## Methods

### Definition of the MOA hypothesis

**Figure 1.**
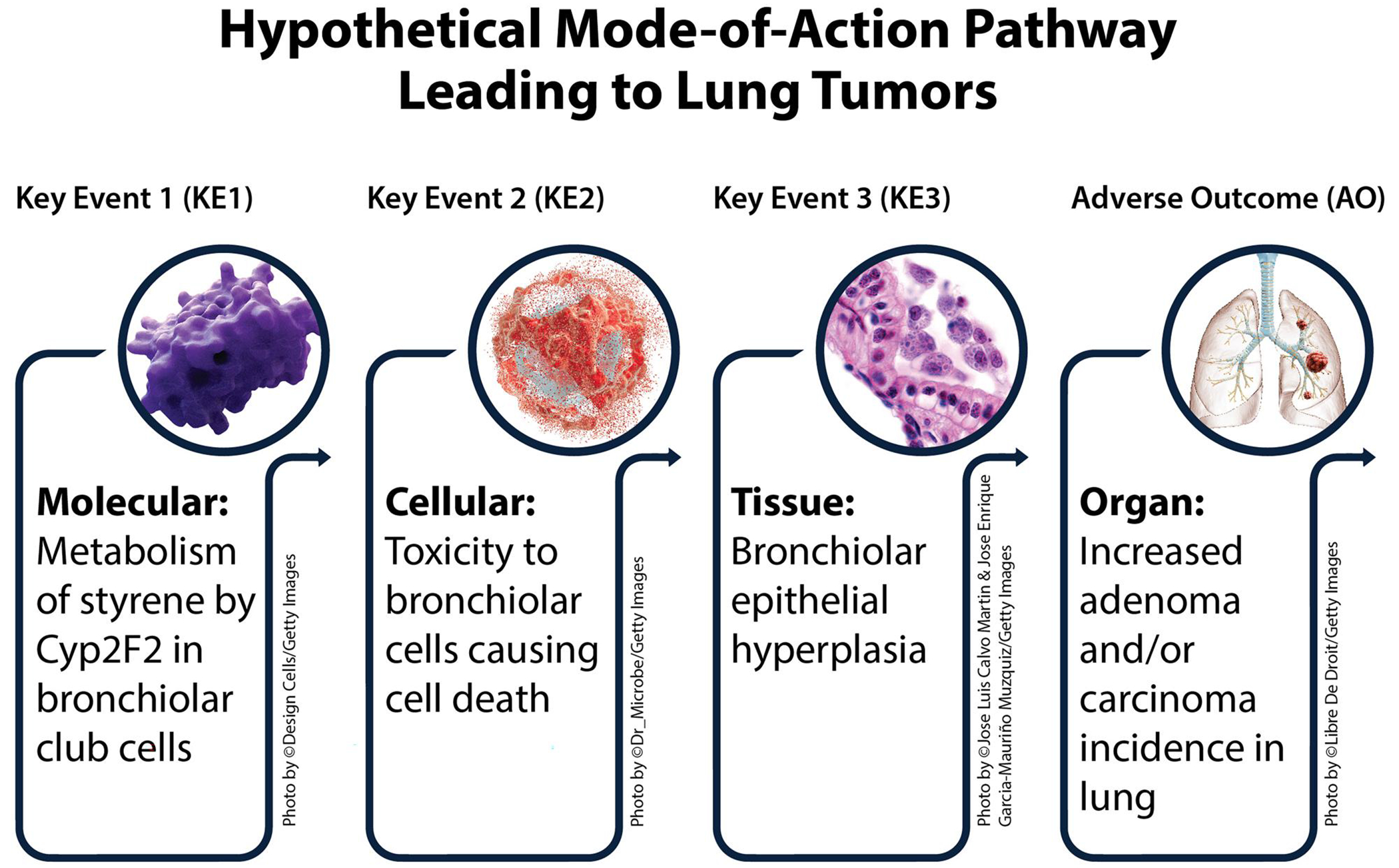
Hypothetical Mode-of-Action pathway leading to lung tumors.

For the purposes of this assessment, the MOA is defined by three observable key events beginning with molecular interaction between Cyp2F2 and styrene in club cells and resulting in the adverse outcome. The hypothesized key events are depicted in Figure 1.

### Primary literature considered in the assessment

Given that the relevant literature concerning styrene has been extensively reviewed by recent authoritative publications, the current assessment employed a narrative rather than a systematic search to compile relevant data. The IARC styrene monograph (Kogevinas et al. 2018) was used to identify animal carcinogenicity and chronic toxicity studies for styrene. In addition, preexisting critical reviews including Cruzan et al. (2018) and Hill and Conolly (2019) were reviewed to identify relevant datasets in the literature.

To probe the literature for any previously uncited data informing the MOA for styrene, Pubmed/MEDLINE and Scopus were searched using the terms in Table 1 with no limitations on date range. No relevant datasets not already identified were found in the literature search.

### Data on key events in the hypothesized MOA identified in literature

The body of literature analyzed for this assessment includes studies in mice and rats exposed to styrene through inhalation and gavage routes. Identified key events consisted of histological lesions of the lungs and airway occurring at time points ranging from 24h to 2years following (repeated) exposure. These were observed in the lungs of mice, but not in the lungs of exposed rats in any study. The studies reviewed in this assessment are summarized in Appendix A.

All incidence data for downstream key events in mice have been arranged in Table 2 for reference. These include incidences of bronchiolar cell death, bronchiolar hyperplasia, and bronchiolar adenoma and/or adenocarcinoma in mice exposed to styrene at listed concentrations. Comparing these allows for weighing evidence in support of KERs. For the purposes of this assessment, it is assumed that metabolism of styrene by Cyp2F2 occurs to a similar extent and with similar kinetics in all wild-type mouse strains exposed to a given dose. Cyp2F2 has been annotated in 18 different inbred mouse strains and expression and metabolic function are not expected to vary significantly between mouse strains, so for the purposes of evaluating the KER linking styrene metabolism to bronchiolar cell death it is assumed that styrene metabolism is functionally equivalent and Table 2 does not describe effect size or incidence data of KE1 (metabolism of styrene by Cyp2F2).

### Application of the evolved Bradford Hill considerations

1. Biological Concordance: The MOA proposes that cellular toxicity from active Cyp2F2-mediated metabolites of styrene causes a hyperplastic phenotype that induces or increases the incidence of adenomas and/or carcinomas in the bronchiolar and bronchioloalveolar regions of mice. This hypothesis is supported by biological concordance if there are known mechanisms that would reasonably be expected to result in this sequence of key events in the circumstances observed in the available data. The key event relationships in question are: styrene metabolism by Cyp2F2 leading to cellular toxicity; cellular toxicity in bronchioles leading to tissue hyperplasia, and hyperplasia preceding the increase in tumor incidence.
2. Essentiality: The MOA hypothesis is supported if inducing the loss of Cyp2F2 function in an appropriate experimental setting blocks the later key events and/or adverse outcome. Conversely, the hypothesis would be directly violated by data demonstrating the subsequent key events and adverse outcome occurring in the absence of metabolism by Cyp2F2 in styrene-exposed models.
3. Concordance of Empirical Observations: The adverse outcome is observed between 20 and 40ppm in susceptible mice. The key events preceding this should occur at similar or lower concentrations. Cellular toxicity (necrosis/cell death) should occur at a similar or lower level than bronchiolar hyperplasia. The incidences of these same key events should also occur in decreasing order. Therefore, the incidence of early bronchiolar cell toxicity should be greater than the incidence of later hyperplasia at the same dose, and the incidence of hyperplasia should be proportionally higher than that of the tumors after accounting for background tumor incidence.
4. Consistency: Because the MOA hypothesis is that metabolism of styrene by Cyp2F2 is the key initiating metabolic KE in the development of lung tumors, the expectation is that species that express Cyp2F2 in airways will develop bronchiolar cell toxicity, hyperplasia, and increased lung neoplasms, in that order, following chronic exposure to sufficient levels of styrene. Species lacking Cyp2F2 should not develop lung tumors in response to chronic styrene exposure.
5. Analogy: The MOA hypothesizes that metabolism of styrene by Cyp2F2 initiates the observed lung effects. Cyp2F2 acts on styrene’s aromatic ring and similar ring-containing molecules. By analogy, data from other Cyp2F2 substrates containing similar aromatic ring structures supports the MOA hypothesis if they also cause bronchiolar cell toxicity in mice, followed by hyperplasia and an increase in lung tumors in an order empirically consistent with the hypothesis. To assess support for the MOA by analogy, toxicity data in mice and rats were reviewed for six Cyp2F2-metabolized chemical analogues of styrene: naphthalene, ethylbenzene, coumarin, cumene, α-methylstyrene, and benzofuran. These were chosen from among known Cyp2F2-metabolized styrene analogues based on the availability of relevant toxicity data, mainly from NTP cancer bioassay studies in mice and rats. Data from both chronic inhalation and gavage studies were used to maximize the number of analogues for comparison. Data for these chemicals supports the MOA by analogy when it is concordant with the MOA expectations: that susceptible mouse strains develop bronchiolar toxicity, hyperplasia, and adenomas and/or carcinomas in the hypothesized order of dose, temporality, and incidence; and that rats do not develop these features at most tested dose levels (if at all).

### Assessment of species concordance

When evidence is sufficient to validate a hypothesized mode of action in an animal model, the next step is a qualitative assessment of whether the hypothesized KERs can or are likely to also occur in humans. Typically, this involves a side-by-side comparison of the MOA events as hypothesized in the animal model directly compared to an analogous key event sequence in humans considering basic differences in anatomy, physiology, endocrinology, genetics, epidemiology, or any other known information regarding the hypothesized KERs as they would plausibly arise in humans. The discussion of species concordance by Boobis et al. (2006), Meek et al. (2003) and Meek, Boobis, et al. (2014) were the primary guidance materials for this step.

Although the MOA is considered in the context of the specific chemical exposure of interest, species concordance does not necessarily evaluate the effects of the chemical in humans. Rather, the MOA itself is evaluated as to whether the KERs established in the animal model are also likely to arise in humans. Because it is the likelihood of the KER being evaluated and not necessarily the effects of the chemical, the data informing these conclusions may or may not be specific to the chemical of interest. In addition to experimental effect data, data used to reasonably exclude the human relevance of individual KERs can include information about the nature, function, and anatomy of the target site, physiological regulatory mechanisms, and the pathogenesis of human and animal disease states across various levels of biological organization.

An MOA that cannot be qualitatively excluded from human relevance may still be excluded based on quantitative differences between the animal model and humans. Quantitative data can take the form of differences in the rates of metabolic activation but may also be incidence data pertaining to a key event or any other form of quantitative data that informs the likelihood of a KER or AO occurring in humans.

## Results

The modified Hill considerations were applied to the data summarized in Table 2 and are addressed in the following sections.

### Biological concordance

The broader biological understanding of the hypothesized MOA is incomplete. The mechanisms causing cell toxicity and death following metabolism of styrene by Cyp2F2 are hypothesized but unknown, and the mechanistic relationship between this damage and the later development of hyperplasia is unclear. Bronchiolar hyperplasia is regarded as a pre-neoplastic lesion in the mouse, so the hypothesized relationship to the outcome is consistent with broader knowledge on this point.

KE1→KE2: Biological knowledge is missing for the relationship between Cyp2F2 activity and cellular toxicity, but the proposed relationship is not implausible. The toxicologically active metabolite(s) of styrene are not known, nor are the mechanism(s) that mediate their effect on bronchiolar cells. Some investigators have hypothesized that Cyp2F2-mediated metabolites of styrene form adducts with protein targets that cause cell death and proliferative responses in bronchioles (Cruzan et al. 2018). Hill and Conolly (2019) also suggest that cellular glutathione (GSH) depletion may play a moderating role.

KE2→KE3: Although there appears to be a link between the cellular toxicity and death induced by styrene and later hyperplastic growth, the mechanisms constituting that link are unknown. Short-term experiments in mice showed that the proliferative response measured in the bronchioles following toxic exposure was short-lived even as repeated exposures continued, and that the bronchiolar epithelium resolved to a flattened, crowded appearance (Green et al. 2001; Andersen et al. 2017). Although active cell replication in bronchioles is robustly induced following styrene-induced cell loss, Green et al. (2001) reported that cell replication (measured by Brd-U labeling) returned to baseline levels within 6days after repeated exposure to 40ppm styrene in male and female CD-1 mice. It is possible that the flattened, crowded bronchiolar epithelium reflects a pre-disease state lacking normal cell functions that play a part in minimizing later tumor growth. For example, Hill and Conolly (2019) suggest that loss of CCSP (club cell secretory protein/CC10) signaling in chronically exposed mice may bias the tissue toward hyperplasia.

KE3→AO: The proposed key event relationship between bronchiolar hyperplasia and the promotion of tumor development is consistent with broader biological knowledge. In humans, uncomplicated hyperplasia is not regarded as a pre-neoplastic lesion (Greenberg et al. 2002), but in the rodent lung, hyperplasia of bronchiolar and alveolar duct epithelium can develop into adenomatous tumors (Renne et al. 2009). In addition, difficulty often exists in consistently characterizing lesions along the morphological continuum of bronchiolar hyperplasia, bronchiolization of the alveolar ducts, adenoma, and adenocarcinoma in the mouse (Renne et al. 2009). This seems to be reflected in the literature reviewed for this assessment, in which description of adenomas versus carcinomas induced by styrene in the lungs of mice varied in key reports (see Cruzan et al. 2001, 2017). Therefore, it is biologically plausible and consistent with the available data that bronchiolar hyperplasia is a key event leading to styrene-induced lung tumors in mice.

### Essentiality of metabolism of styrene by Cyp2F2

The proposed MOA hypothesizes metabolism by Cyp2F2 as the specific molecular event initiating styrene toxicity and its proposed disease process in mice. Therefore, evidence should show that specific inhibition or deletion of Cyp2F2 precludes or impacts the development of the subsequent key events and adverse outcome. Cruzan et al. (2017) show that knockout of Cyp2F2 in male C57Bl/6 mice abrogates virtually all detectable toxicologic responses to styrene in C57Bl/6, building upon a group of related studies characterizing this strain (Cruzan et al. 2012, 2013). These include the key events of bronchiolar cell death and hyperplasia. This is considered direct evidence of essentiality because the experimental manipulation was very specific to Cyp2F2, and therefore there is no ambiguity that the observed differences are due to Cyp2F2 expression and not another factor. In addition, global profiling of whole lung tissue in styrene- vs air-exposed mice by Andersen et al. (2018) showed that styrene induced virtually no early changes in whole lung gene expression when using a 1.5-fold change threshold in the Cyp2F2-deficient C57Bl/6 models, further indicating that early key event responses to styrene are dependent on Cyp2F2 and not on any alternative key event element.

The evidence that Cyp2F2 deletion abrogates the actual tumor response to styrene is indirect because Cyp2F2 knockout mice are only available in the C57Bl/6 strain used in the Cruzan et al. experiments. This strain is naturally resistant to the development of bronchiolar tumors in response to styrene. Therefore, the strain cannot be used to directly demonstrate that the tumor response is dependent on metabolism of styrene by Cyp2F2. Styrene still induces bronchiolar toxicity and hyperplasia in C57Bl/6, and because this kind of tissue change is well known as a precursor lesion to bronchiolar adenoma/carcinoma in rodents (see Biological Concordance above), it is anticipated that these lesions would ultimately progress to tumors in susceptible strains. Because Cyp2F2 knockout models are not available in a tumor-susceptible strain and Cyp2F2 essentiality can only be demonstrated for the early key events, the evidence for the hypothesis is indirect.

There is also no evidence that directly contradicts the hypothesis that Cyp2F2 is essential in the styrene-induced tumor response. Direct counterfactual data would take the form of styrene-induced tumors occurring in exposed animals despite Cyp2F2 inhibition or knockout. A summary of the best evidence supporting the essentiality of the MOA is given in Table 3.

### Concordance of empirical observations

Comparison of the available exposure-response relationships of key events and outcomes in mice supports the hypothesized key event order. Incidence data for key events and the adverse outcome are available from Cruzan et al. (2001) and Andersen et al. (2017) using CD-1 and C57Bl/6 mice, which do not differ in Cyp2F2 expression and are expected to have similar sensitivity to styrene toxicity (see Table 4). The lowest effect concentrations observed for key events in male mice are presented in Table 4 and support the hypothesized key event narrative because the incidences of key events decrease when viewed in order from left to right at a given concentration. Concordantly, earlier key events are first observed at lower exposure concentrations compared to later ones. At 20ppm, in the middle of the dose-effect range, the incidence in male CD-1 mice with hyperplasia is approaching maximal (8/10 animals) while the tumor incidence increases from 15/50 to 21/50 before reaching significance (and maximal effect) at 40ppm.

To further examine the relationship between concentration and key event incidence, we modeled the incidence data for key events in both male and female mice using Benchmark Dose Software (BMDS 3.2 Build 1, www.epa.gov/bmds). Benchmark doses (BMD) were determined corresponding to the central tendency of benchmark responses of 10%, 20%, and 50% extra risk. The modeling analysis included all key event incidence data that were amenable for modeling and utilized a suite of restricted and unrestricted models. Model averaging was considered, but individual models provided the best fit options for the desired benchmark response levels. The models with the best combination of best visual fit, high p value, and low scaled residual values were chosen to determine the BMDs for individual datasets. BMD values are given in Table 5.

Key event data for male and female mice were modeled for bronchiolar cell death (in males), bronchiolar epithelial hyperplasia at 52, 78, and 104weeks, and bronchiolar adenoma (carcinoma incidences could not be modeled). Cruzan (Cruzan et al. 1998) observed focal crowding of bronchiolar epithelium in CD-1 mice exposed to up to 200ppm styrene for 13weeks, but the incidences were not sufficiently increased to be amenable to modeling. The incidence of bronchiolar hyperplasia at 52weeks in female CD-1 mice was also too low for modeling, and the BMD levels of this endpoint in males are high relative to the rest of the matrix. As discussed for biological concordance, the process underlying the temporal development of hyperplasia is not clear, but by 78weeks, a clear ordering of BMD concentrations corresponding to the key event order is apparent, and the dose-incidence profile for bronchiolar hyperplasia at 78 and 104weeks resembles that of bronchiolar adenoma. The BMD concentrations for hyperplasia at 78 and 104weeks are comparable in females, while males were around twice as sensitive at 78weeks vs 104weeks. All BMD values for bronchiolar hyperplasia were lower than the corresponding BMD for bronchiolar adenoma, consistent with the proposed key event order.

### Consistency

Rats do not express Cyp2F2, the closest ortholog in the rat being Cyp2F4. Three studies investigated the effects of chronic styrene exposure in Fisher 443, Sprague-Dawley, and BD IV rats. Male and female Fisher 443 rats were unresponsive to oral styrene doses up to 2000 mg/kg dosed 5days/wk in a 2-yr study (NCI 1979). There were no changes in BD IV rats after 2-year exposures in males and females to 500mg/kg, but the doses were only given on a weekly basis (Ponomarkov and Tomatis 1978). Male and female Sprague-Dawley (CD-1) rats did not respond to up to 1000ppm styrene when exposed for 5d/wk over a 2-yr period (Cruzan et al. 1998). These observations are consistent with the hypothesis that species that do not express Cyp2F2 are not susceptible to styrene toxicity in the lung. CD-1 (Sprague-Dawley) rats were also resistant to the short-term bronchiolar toxicity of styrene following 6h exposures of up to 500ppm for up to five days (Gamer et al. 2004).

All mouse strains tested in the literature reviewed demonstrated early key event toxicity in response to styrene. When tested in chronic bioassays, CD-1 and O20 mice develop lung tumors in response to styrene, while C57Bl/6 does not. B6C3F1 mice (a first-generation hybrid of C3H/He and tumor-resistant C57Bl/6) exhibit no background tumors in the lung but tumors were reported in styrene-exposed B6C3F1 mice following a 13-week recovery period following 78weeks of exposure, although the incidence did not achieve statistical significance (NCI, 1979). If the hypothesized MOA is correct, then strains with contrasting outcomes should show relative differences in Cyp2F2 activity and early key events. Andersen et al. (2017) found that the levels of Cyp2F2 transcripts in lungs from control group C57Bl/6 and CD-1 mice were comparable. Cruzan et al. (2017) found that the extent and severity of Cyp2F2-dependent early and late lesions (bronchiolar degeneration and hyperplasia) were comparable throughout the full 2-yr exposure of C57Bl/6 and CD-1 to 120ppm styrene. These limited data suggest that Cyp2F2 activity and early key events hypothesized in the MOA are not different among strains that do and do not express the adverse outcome. This is not consistent with the hypothesis that Cyp2F2 expression alone determines susceptibility to the tumor response. However, it may point to metabolism by Cyp2F2 being necessary to cause the tumors but not sufficient as a factor by itself when compared across different strains and species.

C57Bl/6 is known to be resistant to a range of tumors common in the mouse (https://www.jax.org/strain/000664), so it may be the case that this specific trait interacts with the adverse outcome but not the earlier key events. The strains with the highest tumor susceptibility (CD-1, O20) also had high background incidences of bronchiolar tumors (Ponomarkov and Tomatis 1978; Cruzan et al. 1998). C57Bl/6 did not exhibit background tumors or susceptibility to styrene-induced tumors. Bronchiolar tumors are not a common background lesion in the rat, which is also not susceptible to styrene-induced lung tumors (Cruzan et al. 1998). The bronchiolar tumor response in mice could represent a risk of styrene exposure that is limited to tumor-susceptible subtypes that may not extend beyond the mouse.

### Analogy

Out of the six analogues, four were ultimately concordant for the intermediate KEs and adverse outcome (see Table 6). Most of the studies, including all 2-yr cancer bioassays, were conducted in B6C3F1 mice and F344/N rats. Naphthalene, ethylbenzene, coumarin, cumene, and benzofuran increased the incidence of adenomas and/or carcinomas in the alveolar/bronchiolar region, while no lung tumors were observed in rats. Of these, ethylbenzene, cumene, and benzofuran, and naphthalene were fully concordant with respect to intermediate key events, with bronchiolar hyperplasia being exhibited in the expected order of dose-response and incidence. The remaining analogues exhibited some degree of variation from expectations, with coumarin being the only dataset that was discordant. Because the discrete mechanisms of the toxic effects of Cyp2F2-metabolites in mouse club cells are not known, it is possible that some analogues examined may exhibit toxic qualities that deviate from some of the expectations of the MOA. For the purposes of evaluating the MOA evidence for styrene specifically, the WOE by analogy considers the trend of analogue toxicity as a group.

Naphthalene was concordant with the MOA in terms of bronchiolar injury and tumorigenicity in mice compared to rats (NTP 2000). The observations in mice in this study were somewhat inconsistent with the MOA hypothesis. Hyperplasia of the respiratory epithelium and chronic inflammation were observed at the 2-year time point in almost all animals of both 10 and 30ppm groups in both sexes, but only female mice developed bronchiolar tumors. The expectation is that both sexes would develop tumors because both sexes developed maximal levels of hyperplasia, particularly since males are more sensitive to styrene across a range of previous datasets. This data is not contradictory to the MOA hypothesis, but it does not lend direct support because it does not demonstrate the MOA outcome in this case.

Coumarin caused necrosis of bronchiolar cells in female B6C3F1 mice 1 day after a 200 mg/kg *via* gavage, but no interim hyperplasia was seen in chronic studies at that daily dose level in male or female mice when examined at 13 wks, 15months, and 2 years (NTP 1993; Born et al. 1998). Coumarin did moderately increase bronchiolar adenoma/carcinoma incidence in male mice from 14/50 affected in vehicle controls at 2 yrs to 24/51 in mice exposed to 200mg/kg/d *via* gavage, but hyperplastic lesions were not significantly increased. This is not consistent with how the key event narrative presents in CD-1 mice exposed to styrene, where the incidence of hyperplasia meets or exceeds that of lung tumors at the end of the 2yr assay (Table 2). These data are considered in the context of the other analogous data examples. It can be noted that the highest dose tested in the coumarin study appears to be near the threshold of adverse effect for coumarin. Marginal differences in the interpretation of pathology may impact the findings in this range.

α-Methylstyrene was the only analog that failed to induce the MOA in mice, resulting in no bronchiolar lesions or lung tumors when examined at 13 weeks and 2 years of exposure to up to 600ppm (NTP 2007). Because the toxic metabolite of styrene is not known, it is also not possible to rule out that some analogues will not form the cytotoxic moiety. It is also possible that the concentrations used in this study were not high enough to cause toxicity if that moiety is formed. The potency of other analogues in causing those effects varies widely. Therefore, the overall weight of evidence by way of analogous examples is not counterfactual to the hypothesized MOA and key event sequence, and data for a clear majority of examples support the MOA.

### Species concordance

This section evaluates the likelihood that the KERs that make up the MOA identified in mice would also arise in humans based on qualitative and quantitative comparisons between the biological systems. The key events and relationships discussed here have been rephrased somewhat from their origins in the mouse model in order to better translate to an essentially analogous sequence that could hypothetically arise in humans. For example, KE1 is rephrased as ‘conversion to toxic metabolites’ instead of ‘Cyp2F2-mediated metabolites’ because humans do not express the mouse enzyme but could be producing other enzymes that have a similar functional result.

### Concordance of KE1: conversion of styrene to one or more cytotoxic metabolite(s)

In the mouse model, it is hypothesized that Cyp2F2 converts styrene to one or more cytotoxic metabolites in the lung. The exact identity of the metabolite(s) is unknown, so it is difficult to ascertain whether enzymes expressed in the human lung could produce a qualitatively similar outcome. Carlson (2008) reviews the data informing the metabolism of styrene in the human lung, for which the CYP2F1 enzyme is the most likely mediator based on *in vitro* oxidation studies by Nakajima et al. (1994) and structural considerations. There is very little direct qualitative data regarding CYP2F1 beyond the Nakajima et al. (1994) study measuring styrene glycol formation *in vitro*, which was only marginally higher than mouse Cyp1A1. Expression of the human CYP2F1 enzyme in transgenic Cyp2F2-null mice failed to effectuate the acute cytotoxicity of styrene exposure (Andersen et al. 2017), suggesting that the metabolic potency of the human enzyme itself is not comparable to rodent Cyp2F enzymes. In the mouse lung, Cyp2F2 efficiently generates both styrene oxide and ring-oxidized products that include 4-vinylphenol (4-hydroxystyrene). Experimental work by Carlson (2002) indicated that ring-oxidized metabolites are the toxic products in mice, and require multiple metabolic steps mediated by Cyp2F2. In humans, ring oxidation is a very minor pathway accounts for between zero and 1% of the total excreted dose of absorbed styrene when measured experimentally (Johanson et al. 2000, Vodicka et al. 2006).

Although not knowing the identity of the toxic metabolite(s) obstructs a thorough qualitative assessment, some quantitative comparisons can be made between mice and humans. Experimental analyses using lung microsomal preparations indicated that styrene is metabolized to styrene oxide hundreds of times faster in the mouse lung than human (Carlson, 1997; Csanâdy et al. 2003). The pharmacokinetic model used by Sarangapani et al. (2002) also predicted roughly a 100-fold difference in the concentration of styrene oxide in the bronchioles of mice vs humans. In *ex vivo* studies using nasal tissue, styrene oxide was efficiently formed by mouse nasal explants but below the level of detection in human (Green et al. 2001). Although the available data do not necessarily indicate that styrene oxide is the toxic metabolite (Cruzan et al. 2012), it is a good index of the rate of styrene metabolism by Cyp2F2 in the mouse.

While it is not possible to fully exclude the plausibility of styrene’s toxic metabolite being produced in the human lung, the broader biological knowledge speaks against this. The above data are consistent with a number of other cases recently reviewed in Oesch et al. (2019) of chemical susceptibilities in the mouse caused by much higher rates of metabolic activity in the lung compared to human. These include naphthalene, which is metabolized at a much higher rate by Cyp2F2 when compared to human CYP2F1 (Shultz et al. 1999).

### Concordance of KE2: airway cell death following xenobiotic metabolism

As reviewed by Pandiri (2015), rodents and mice in particular have a combination of features that make them relatively susceptible to chemically-induced bronchiolar injury. Generally, this is due to the morphology and cell composition of the respiratory tract, in which non-ciliated club cells make up 75% of the bronchiolar epithelium by number in rats and 90% in mice. By comparison, the composition of human bronchiolar epithelium varies from 11-40% club cells (Plopper and Hyde 2015). Rodents and mice in particular are thus more susceptible to chemically induced injury because of the greater metabolic capacity of the small airways compared to human airways. This is a known qualitative difference between rodents and humans (Pandiri 2015), but data do not exist to compare the susceptibility of club cells in quantitative terms.

### Concordance of KE3: hyperplasia of airway epithelium following cell death

Hyperplastic foci occurring in the terminal bronchioles and bronchioloalveolar region are common following long-term repeated toxic injury in mice (Renne et al. 2009). These effects are well-characterized in rodents and are the most common chemically-induced non-neoplastic lung lesion in mice (Dixon et al. 2008). Airway hyperplasia is not a well-characterized event in humans. Pandiri (2015) notes that atypical adenomatous hyperplasia (AAH) is the human lesion most analogous to bronchiolar hyperplasia in rodents. AAH are small proliferative foci consisting of a single layer of atypical club-like cells lining bronchioloalveolar and alveolar spaces. These cells are atypical in size, nuclear morphology, and generally disarrayed appearance (Mori et al. 2001). While hyperplasia is a well-known response to repeated toxic injury in the mouse lung, it is not known whether AAH develops as a result of repeated damage in humans. If it does, data describing the rate and sensitivity of the hyperplastic response following airway cytotoxicity may allow for a quantitative comparison to the mouse.

### Concordance of the AO: increased incidence of lung tumors and cancer

Dixon et al. (2008) summarize that alveolar/bronchiolar hyperplasia and bronchiolar adenoma/carcinoma are the most common chemically-induced non-neoplastic and neoplastic lesions, respectively, in both mice and rats. The progression from distal airway hyperplasia to adenoma and adenocarcinoma is well-known in mice, but there is debate on the subject of whether lung tumors arising in mice are generally of relevance to human lung tumors (Renne et al. 2009). Hahn et al. (2007) noted that at the time of publication, 15 out of 30 human lung carcinogens known to IARC had been tested in rat and mouse cancer bioassays. Eleven and 5 out of the 15 carcinogens produced lung tumors in rats and mice, respectively.

While there are some histological and morphological similarities between lung tumors in mice and humans, there are some substantial differences that speak to a lack of biological concordance, particularly for environmental cancers. These differences and their implications for health policy have been reviewed by Nikitin et al. (2004) and Cohen et al. (2020). The bronchioloalveolar tumors that are well-recognized in mice are generally homogenous in terms of histology and noninvasive, while human lung carcinomas tend to be heterogenous or of mixed subtype and are generally invasive. While peripheral adenomatous tumors are common in mice, the majority of human airway tumors arise from the bronchi and are related to smoking, including non-small cell cancers. In nonsmokers, peripheral lung adenocarcinomas do arise and bear some substantial similarities to adenocarcinomas in mice in terms of morphological features and transcriptional profiles (Pandiri et al. 2012; Pandiri 2015). These features make comparison possible although the functional analogy between the two species is unclear.

The nomenclature for human lung tumors arising in the peripheral airways has been evolving to delineate histological and site-specific features of prognostic significance. These developments are reviewed in detail by Raz et al. (2006), Travis et al. (2011), Xu et al. (2012), and Nicholson et al. (2022). Tumors arising in the bronchioloalveolar region in humans are currently differentiated as mucinous or non-mucinous. Non-mucinous tumors resembling those arising in mice typically exhibit lepidic growth with preserved alveolar structure and are classified as either adenocarcinoma in-situ (AIS) or minimally-invasive adenocarcinoma (MIA). AIS comprises cells resembling club cells or alveolar type II cells, the distinction being deemed clinically insignificant (Travis et al. 2011). Both AIS and MIA have a favorable prognosis in humans.

Based on a simple comparison of site occurrence, histology, and prognostic significance, AIS is the human lesion that most resembles the bronchioloalveolar tumors observed in mice. The relationship between the lepidic patterns of AAH and AIS are similar to that of the bronchioloalveolar hyperplasia and lung tumors in the mouse, and examination of molecular biomarkers also support AAH as a precursor lesion to AIS (Nicholson et al. 2022). It is unclear whether these features represent a functional analogue to the chemically-induced airway tumors in mice. There is no indication that progression from AAH to AIS occurs as a consequence of repeated airway injury in humans, but this would be difficult to capture in the form of data.

Based on a comparison of homology, mucinous adenocarcinoma in humans most frequently carries mutations in the *KRAS* gene region that is homologous to the *PasI* susceptibility locus in tumor-prone mice such as CD-1 (Manenti and Dragani 2005). Individual cases of non-mucinous AIS and MIA subtypes can exhibit mutations in either *KRAS* or *EGFR* genes, or may present with neither of these (Travis et al. 2011). These mutations occur early in the hyperplastic disease process and do not typically occur together (Nicholson et al. 2022). From the current data, it is not clear whether a particular genetic subtype of peripheral human lung tumor represents a functional analogy to those in mice.

There is little epidemiological observation of lung cancers associated with styrene exposure in humans. A working group for the National Research Council (NRC 2014) concluded that there was no significant evidence that styrene exposure was associated with lung cancers in a review of several occupational epidemiology studies. Occupational exposures to styrene in epidemiological studies are often complicated by co-exposure to 1,3-butadiene, however Bertke et al. (2018) and Daniels and Bertke (2020) were able to identify occupational cohorts without this interference. These studies found a slight excess of lung cancer mortality risk among styrene-exposed workers, but the authors concluded data did not support a causal relationship between styrene exposure and lung cancer. An additional analysis of the data suggested that healthy-worker survivor bias could obscure a possible positive association in these studies Bertke et al. (2021).

## Conclusions

### Does the weight of evidence support the proposed MOA in mice?

A summary of the direct, indirect, contradictory, and missing evidence for the evolved Bradford-Hill considerations for the hypothesized MOA is presented in Table 7. The Cyp2F2-dependent MOA hypothesis is directly supported by dose-response relationships between observed key events and the adverse outcome, and there is no evidence directly contradicting the hypothesis that metabolism by Cyp2F2 is necessary and causal in bronchiolar tumors observed in susceptible mouse strains. There is direct support that metabolism by Cyp2F2 is essential for the observed interim key events, and it is biologically concordant that the interim events would lead to the adverse outcome.

The MOA hypothesis is consistent with regard to the key event observations being restricted to Cyp2F2-expressing systems, but the observations are not consistent with the hypothesis that metabolism by Cyp2F2 is sufficient on its own to cause the outcome. If this were true, mouse strains with similar expression of Cyp2F2 should have similar outcomes. Because susceptibility to the tumorigenic effects of styrene varies between strains of mice that similarly express Cyp2F2, it is evident that Cyp2F2 expression alone does not determine whether styrene exposure will result in bronchiolar tumors in another test system. This does not contradict that Cyp2F2 may be necessary for the outcome to occur, even in susceptible systems.

The only evidence that is directly inconsistent with the proposed key event pathway is analogous data for coumarin. Coumarin causes early bronchiolar cell toxicity and increases tumor incidence in B6C3F1 mice, but interim hyperplasia was not observed in bronchioles in the NTP cancer bioassay (NTP 1993). Although this is not consistent with the hypothesis of bronchiolar hyperplasia being a key event, this can be taken into consideration with the fact that the increase of bronchiolar tumors in mice in this case appears to be at the limits of detectability and is only detectable in males, and also that the majority of analogous evidence is concordant with hypothesized key event order.

Based on the weight-of-evidence from the best available datasets, it is likely that metabolism by Cyp2F2 is necessary, but not sufficient alone to cause or increase the incidence of bronchiolar tumors in mice exposed to styrene. Significant impact on tumor incidence is seen in mice with high levels of background tumors, but not all strains of mice share this phenotype.

### Is the MOA proposed in mice likely to arise in humans?

Because the toxic metabolites of styrene and their mechanism(s) of action are not known, it is not possible to exclude the MOA from human relevance on qualitative grounds, but a comparison of mouse and human systems speaks against this. While cells in the human airways are capable of producing most of same styrene metabolites as in the mouse, the capacity for styrene metabolism by any conceivable means is so much lower in the human lung that it is unlikely that the concentration of any metabolite would reach a level causing frank cytotoxic effects. If cytotoxicity were to occur as it does in the mouse, it is not known whether an analogous hyperplastic or proliferative process would follow in the human lung. The continuum of hyperplastic bronchiolar lesions progressing to adenoma and carcinoma in rodents generally bears poor histological and site concordance with human lung lesions, especially when considering chemically induced tumors. This aligns with the broader observation that mice are susceptible to a range of xenobiotic lung carcinogens on the basis of metabolic capacity. These comparisons are summarized with supporting data in Table 8.

## Discussion

The weight-of-evidence indicates that it is very likely that metabolism by Cyp2F2 is a necessary key event mediating bronchiolar tumor responses in mice after chronic styrene exposure, and that the adverse outcome is further limited to susceptible genetic backgrounds among mice. It is reasonable to conclude these tumors do not reflect an analogous tumor hazard in humans exposed to styrene. This is underscored by weak concordance of key event plausibility between mice and humans in both early and later key events. This conclusion does not preclude the possibility that styrene could cause lung cancer in humans through an entirely unrelated pathway, but to date there is no indication that it does so.

Pandiri (2015) notes that expectations of site concordance of tumors between species are never absolute and instead emphasizes identifying common pathways of disease in both species. Given that the majority of toxicological lung cancers in humans arise from the bronchi and central airways, a more complete understanding of the species concordance of styrene’s effects on mice would be gained by data specific to styrene metabolism and toxicity in the central airway cells from which most human cancers are understood to arise. Comparison of the xenobiotic responses between human bronchial airway and murine terminal bronchioles would shed light on remaining questions about when and how environmentally-induced peripheral adenocarcinomas seen in cancer bioassays can reflect analogous disease processes in humans. Currently available exposure systems using primary human airway cells grown at an air-liquid interface may be well-suited to such work.

### Toxic mechanisms of club cell death and the hyperplastic phenotype

The key events hypothesized in the MOA were defined based on known biology and direct observation of events manifested in available experimental data. It is still unclear what the exact mechanisms mediating the KERs are, particularly the cellular events immediately following metabolism of styrene by Cyp2F2. Two other critical reviews of this topic have proposed different possibilities. Cruzan et al. (2018) and Andersen et al. (2017) suggested styrene metabolites interact with unknown nuclear proteins to produce a mitogenic signal driving proliferative effects, hyperplasia, and tumors, with cell death being an incidental rather than key event. In another review, Hill and Conolly (2019) proposed that formation of protein adducts and increase in oxidative stress led to loss of club cells and/or the CCSP-expressing club cell phenotype, creating a hyperplastic environment. In either hypothesis, cellular events immediately following styrene metabolism are largely speculative as experimental data at the level of the club cell microenvironment are limited. However, the ambiguity of exact mechanisms does not preclude application of the human relevance framework. Because bronchiolar cell death has strong incidence and dose concordance with later key events, it is likely to be a necessary key event although it is not possible to rule out contributions from a direct mitogenic mechanism.

A limitation in the body of literature is the lack of a test system that can directly test the proposed essentiality of metabolism by Cyp2F2 as a necessary key event limiting the adverse tumor response to the mouse. Genetic knock-out mouse models are clearly the best choice to demonstrate essentiality, but the popular C57Bl/6 background is unsuitable for testing some tumor outcomes because of the strain’s naturally low incidence of spontaneous lung tumors and resistance to many lung tumor types related to distribution of the *PasI* locus (Manenti and Dragani 2005). A gene knockout model (or suitable inhibition or knockdown model) in a more susceptible strain may provide for direct evidence.

### Plausibility of alternative modes of action

The IARC monograph on styrene extensively reviewed key characteristics and mechanisms related to chemical carcinogenesis, concluding that styrene is a possible human carcinogen based in part on strong evidence of proliferative, receptor-mediated, and genotoxic effects thought to be caused by not only styrene metabolites, but styrene itself as well (Kogevinas et al. 2018). Accordingly, these aspects lead the consideration of Cyp2F2-indpendent pathways that could plausibly produce the lung tumor responses seen in mice and are discussed below.

Alteration of cell proliferation by styrene and/or metabolites was observed in a variety of human and non-human systems that included human lymphocytes and rat forestomach (Kogevinas et al. 2018). However, the majority of the data reviewed by IARC demonstrating alteration of cell proliferation is obtained from the same mouse models that have been under examination in the current assessment, in which all proliferative effects detected were dependent on metabolism by Cyp2F2. Because data already show that styrene’s proliferative effects in the mouse lung are Cyp2F2-dependent, an alternative MOA based on cellular proliferative effects of the parent compound or activation by a different enzyme can be excluded.

Receptor-mediated effects of styrene reviewed by IARC are based on data from environmentally and occupationally exposed human cohorts that showed increased levels of serum prolactin (Kogevinas et al. 2018). Elevated prolactin is not known to affect development of any tumor types aside from a modest association with breast cancer and there is no biological knowledge supporting a connection to the incidence of any kinds of lung tumors (Tikk et al. 2015). Furthermore, these data were obtained from human exposures, and there are no human cancers that have been positively associated with these exposures (Daniels and Bertke 2020). It is therefore not anticipated that these kinds of potential hormonal effects play any role in lung tumors analogous to those in the mouse.

IARC state that “there is *strong* evidence that both styrene and styrene-7,8-oxide are genotoxic, and this mechanism can also operate in humans” (Kogevinas et al. 2018). A potential MOA based on Cyp2F2-independent genotoxic effects is considered in the mouse based on concordance with dose-response, consistency, and species differences. There are four studies in mice reviewed in the IARC Monograph examining DNA adduct formation in the lung after styrene inhalation exposures that ranged from 160 to 350ppm. One study (Vodicka et al. 2001) used a multi-dose format that measured styrene-derived DNA adducts one day following 6h exposures, finding the dose response for adduct formation increases substantially between 176 and 350ppm. This is not concordant with the dose-response for lung tumors that appear to reach maximum response at 40ppm. Another study did not detect DNA adducts in mouse lungs following exposure to 40 or 160ppm styrene for 14d (Otteneder et al. 2002). These data speak against dose-response concordance considering a genotoxic MOA. Furthermore, Boogaard et al. (2000) found that DNA adducts measured in isolated mouse club cells immediately following tumorigenic exposure to styrene were not substantially different from the total lung, suggesting that DNA reactivity is not an early key event in the established target cell subpopulation. Finally, comparisons to rat lung carried out in the Otteneder et al. and Boogaard et al. studies show that overall adduct formation following tumorigenic styrene exposures are similar in both rat and mouse. These data indicate that a genotoxic MOA is both non-concordant with dose-response data and inconsistent with expected differences between target tissues and species.

In terms of species concordance between mice and humans, a hypothetical MOA initiated by a genotoxic event is unlikely to be relevant to humans for similar reasons as the original Cyp2F2 hypothesis. Although styrene is weakly genotoxic on its own, at least 95% of the relative genotoxic potency of absorbed styrene is due to styrene oxide and it is accepted that, for practical purposes, genotoxic effects of styrene *in vivo* require metabolic activation to oxides (Kogevinas et al. 2018). Vodicka et al. (2001) also note that further metabolism to of styrene oxide to non-genotoxic styrene glycol by epoxide hydrolase is almost immediate in human lung under normal physiological conditions. Because styrene metabolism to any kind of oxide in humans is minimal relative to the mouse, and metabolites that are generated are readily detoxified, the quantitative difference between the level of potentially genotoxic styrene metabolites in human lung compared to the mouse at any time of exposure speaks against any such MOA being relevant.

### Temporal mapping of the MOA

Although there is good empirical support for the proposed key event pathway and the events occur in the correct order, there are open questions about the details of the disease process and when they occur over the 2-year exposure period. Bronchiolar tumors in susceptible strains of mice are only observed after two years of repeated styrene exposure, which is considered the lifetime of the animal. Exposure typically begins at 7-8 weeks of age. Necrosis develops between 6 and 24 h in male mice, followed by a brief increase in proliferation rates (Green et al. 2001; Andersen et al. 2017). By the second week of exposure, labeling indices for proliferation in bronchioles return to baseline (Cruzan et al. 1997). Focal crowding is evident in bronchioles in the weeks following the start of exposure. Significant hyperplasia is not seen in CD-1 mice until 78 weeks (Cruzan et al. 2001).

Because the exact details of the mechanisms mediating the hyperplastic and tumorigenic effects of styrene metabolism by Cyp2F2 are not known, it is not possible to precisely map the progression of key events over the two-year exposure and observation period. One explanation may be that yet-unidentified intermediate elements make up a continuous pathogenic process that takes two years to fully manifest. A more likely explanation could be that styrene exposure and early key events interact with a life stage-dependent susceptibility occurring in older CD-1 mice and other susceptible strains. In other words, similar tumor outcomes would be observed if the protocol began exposing animals at a later age (e.g. 78 weeks). This hypothesis, while testable, would also require additional research.

## Figures and Tables

**Table 1.** Literature search terms.

Chemical identifiers	100-42-5, styrene, phenylethylene, vinylbenzene, not polystyrene, not nitrostyrene
Keywords	tumors, neoplasm, adenoma, carcinoma, adenocarcinoma, animal, rodent, model, airway, bronchiole, airway, inhalation, oral, exposure, dose

**Table 2.** Incidence data identified in literature for hypothesized key events downstream of metabolism of styrene in mice.

Key Event Endpoint (as described by authors)	Species/Strain/Sex	Exposure	Dose/Concentration					Reference
			Results/Incidence					
KE2: Bronchiolar epithelial cell death	Male C57Bl/6 mice	Whole-body for 6h, endpoint at 24h	0 ppm 0/10	10 ppm 5/10	40 ppm 8/10	120 ppm 8/10		Andersen et al. (2017)
	Male C57Bl/6 (*Cyp2F2*^(−/−)^)mice		0 ppm 0/10	10 ppm 0/10	40 ppm 0/10	120 ppm 0/10		
	Male C57Bl/6 (*Cyp2F2*^(−/−)^/*CYP2F1*^(+/+)^) mice		0 ppm 0/10	10 ppm 0/10	40 ppm 0/10	120 ppm 0/10		
KE3: Focal crowding of bronchiolar epithelium	Male CD-1 mice	Whole-body exposure 6h/d, 5d/wk, for 13 wks	0 ppm 0/10	50 ppm 0/10	100 ppm 3/10	150 ppm 4/10	200 ppm 5/10	Cruzan et al. (1997)
	Female CD-1 mice		0 ppm 0/10	50 ppm 0/10	100 ppm 2/10	150 ppm 2/10	200 ppm 2/10	
KE3: Bronchiolar epithelial hyperplasia	Male CD-1 mice	Whole-body exposure 6h/d, 5d/wk for 52 wks	0 ppm 0/10	20 ppm 0/10	40 ppm 2/9	80 ppm 9/10	160 ppm 10/10	Cruzan et al. (2001)
	Female CD-1 mice		0 ppm 0/10	20 ppm 0/10	40 ppm 4/9	80 ppm 7/10	160 ppm 10/10	
	Male CD-1 mice	Whole-body exposure 6h/d, 5d/wk for 78 wks	0 ppm 0/8	20 ppm 7/10	40 ppm 9/10	80 ppm 8/8	160 ppm 6/6	
	Female CD-1 mice		0 ppm 0/8	20 ppm 7/9	40 ppm 8/8	80 ppm 9/9	160 ppm 10/10	
	Male CD-1 mice	Whole-body exposure 6h/d, 5d/wk for 104 wks	0 ppm 0/50	20 ppm 10/50	40 ppm 37/50	80 ppm 48/50	160 ppm 46/50	
	Female CD-1 mice	Whole-body exposure 6h/d, 5d/wk for 98 wks	0 ppm 0/50	20 ppm 21/50	40 ppm 39/50	80 ppm 45/50	160 ppm 45/50	
KE3: Hyperplasia in terminal bronchioles	Male CD-1 mice	Whole-body exposure 6h/d, 5d/wk for 78 wks^1^	0 ppm 0/5	120 ppm 3/3				Cruzan et al. (2017)
	Male C57Bl/6 mice		0 ppm 0/2	120 ppm 4/5				
	Male C57Bl/6 (*Cyp2F2*^(−/−)^) mice		0 ppm 0/5	120 ppm 0/5				
	Male C57Bl/6 (*Cyp2F2*^(−/−)^/*CYP2F1*^(+/+)^) mice		0 ppm 0/5	120 ppm 0/3				
AO: Combined lung tumors	Male O20 mice	Weekly gavage from birth to 2 yrs	0 mg/kg (oil) 8/19	1350 mg/kg 20/23				Ponomarkov and Tomatis, (1978)
	Female O20 mice	Weekly gavage from birth to 2 yrs	0 mg/kg (oil) 14/21	1350 mg/kg 32/32				
	Male C57Bl/6 mice	Weekly gavage from birth to 2 yrs	0 mg/kg (oil) 3/12	1350 mg/kg 1/24				
	Female C57/Bl6 mice	Weekly gavage from birth to 2 yrs	0 mg/kg (oil) 1/13	1350 mg/kg 1/24				
AO: Bronchiolar/alveolar adenoma (carcinoma)^2^	Male B6C3F1 mice	Gavage 5d/wk with 78 wks with 27 wks recovery	0 mg/kg 0/20	150 mg/kg 3(3)/45	300 mg/kg 4(5)/49			NCI (1979)
	Female B6C3F1 mice	Gavage 5d/wk with 78 wks with 27 wks recovery	0 mg/kg 0/20	150 mg/kg 1/44	300 mg/kg 3/48			
AO: Bronchiolo-alveolar adenoma	Male CD-1 mice	Whole-body exposure 6h/d, 5d/wk for 104 wks	0 ppm 15/50	20 ppm 21/50	40 ppm 35/50	80 ppm 30/50	160 pm 33/50	Cruzan et al. (2001)
	Female CD-1 mice	Whole-body exposure 6h/d, 5d/wk for 98 wks	0 ppm 6/50	20 ppm 16/50	40 ppm 16/50	80 ppm 11/50	160 pm 24/50	
AO: Bronchiolo-alveolar cacinoma	Male CD-1 mice	Whole-body exposure 6h/d, 5d/wk for 104 wks	0 ppm 4/50	20 ppm 5/50	40 ppm 3/50	80 ppm 6/50	160 pm 7/50	
	Female CD-1 mice	Whole-body exposure 6h/d, 5d/wk for 98 wks	0 ppm 0/50	20 ppm 0/50	40 ppm 2/50	80 ppm 0/50	160 pm 7/50	
AO: Bronchiolo-alveolar adenocarcinoma	Male CD-1 mice	Whole-body exposure 6h/d, 5d/wk for 104 wks	0 ppm 7/67	120 ppm 17/67				Cruzan et al. (2017)
	Male C57Bl/6 mice		0 ppm 0/69	120 ppm 0/70				
	Male C57Bl/6 (*Cyp2F2*^(−/−)^) mice		0 ppm 2/69	120 ppm 0/69				
	Male C57Bl/6 (*Cyp2F2*^(−/−)^/*CYP2F1*^(+/+)^) mice		0 ppm 1/69	120 ppm 0/68				

1 Cruzan et al. (2017) collected histological data at 1, 26, 52, 78, and 104 weeks of exposure. Hyperplasia of bronchioles at 78 weeks is shown as representative data while the remaining data are not shown for the sake of organization. The remaining histology data shows bronchiolar cell necrosis at 1, 26, and 104 weeks of exposure in styrene-exposed CD-1 and C57Bl/6 mice and bronchiolar hyperplasia was seen at all interim timepoints. No effects were seen in Cyp2F2-deficient variants.

2 Incidences of carcinoma are noted with parentheses in the Results column.

**Table 3.** Evidence that key events and the adverse outcome in mice are dependent on metabolism of styrene by Cyp2F2.

Event	Direct Evidence	Indirect Evidence	Contradictory Evidence	Missing Evidence
**KE1 Metabolism of styrene by Cyp2F2**	Cyp2F2 is the major pathway for styrene metabolism in mouse lung^1^	None	None	None
**KE2 Bronchiolar cell death**	Cyp2F2 knockout abrogates cell death in C57Bl/6 mice^2,3,4^	None	None	None
**KE3 Bronchiolar hyperplasia**	Cyp2F2 knockout abrogates and styrene-induced hyperplasia in C57Bl/6 mice^2,3,4^	None	None	None
**AO: Bronchiolar adenoma/carcinoma**	None	Bronchiolar hyperplasia (dependent on Cyp2F2) is anticipated to be the precursor lesion to bronchiolar tumors^5^	None	Direct evidence is not available because the only Cyp2F2 KO model is in a tumor-resistant strain that develops early key events but is resistant to a wide variety of lung tumors

This table summarizes the key experimental evidence demonstrating that the events and adverse outcome observed in mice are dependent specifically on metabolism of styrene by Cyp2F2. There is direct evidence demonstrating Cyp2F2 dependency across all interim key events, and indirect evidence that the adverse outcome itself is dependent on Cyp2F2. There is no evidence contradicting the hypothesis.

1 Sarangapani et al. 2002;

2,3,4 Cruzan et al. 2012, 2013, 2017;

5 Renne 2009.

**Table 4.** Dose-response X temporality concordance for Cyp2F2-dependent key events and adverse outcome.

Inhalation Concentration in male mice	KE2: Bronchiolar cell death (6-24h)^1^	KE3: Bronchiolar Hyperplasia^2^	AO: Adenoma/Carcinoma^2^
0 (control)	0/10	0/8 (78 wks)	15/50 (104 wks)
10 ppm	5/10		
20 ppm	8/10	7/10 (78 wks)	21/50 (104 wks)
40 ppm	8/10	9/10 (78 wks)	35/50 (104 wks)

Data in the table are the lowest-observed effect concentrations for the hypothesized key events in male mice. The populated matrix displays a clean left-to-right reading slope in the direction of increasing concentration, indicating that the weight-of-evidence supports the hypothesized sequence of key evidence from the perspective of dose-response and incidence concordance. The incidence data from the lowest-observed effects levels for key events at each available concentration show a left-right reading trend of decreasing incidence at a given concentration. In other words, key events approach maximal effect at the same time or before later key events, consistent with the hypothesized MOA.

1 Andersen et al. (2017);

2 Cruzan et al. (2001).

**Table 5.** Benchmark concentrations for key event responses in mice exposed to styrene.

Male mice	Cell death (24h)	Bronchiolar hyperplasia (52 wks)	Bronchiolar hyperplasia (78 wks)	Bronchiolar hyperplasia (104 wks)	Bronchiolar adenoma (104 wks)	Data Source(s)
BMD_10_ (ppm)	4.6	38	6.1	16	18	Andersen et al. (2017);
BMD_20_ (ppm)	6	39	8.5	20	21	Cruzan et al. (2001)
BMD_50_ (ppm)	10	43	15	29	36	

Female mice	Bronchiolar hyperplasia (78 wks)	Bronchiolar hyperplasia (104 wks)	Bronchiolar adenoma (104 wks)	Reference
BMD_10_ (ppm)	14	10	43	Cruzan et al. (2001)
BMD_20_ (ppm)	16	14	97	
BMD_50_ (ppm)	18	23	281	

**Table 6.** Analogues of styrene and concordance in mouse and rat test systems.

Chemical (CAS #)	Mouse			Rat			
	KE2: Bronchiolar Cell Death	KE3: Bronchiolar Hyperplasia	AO: Tumors	KE2: Bronchiolar Cell Death	KE3: Bronchiolar Hyperplasia	AO: Tumors	Concordance with MOA
Naphthalene (91-20-3)	Cell swelling and necrosis in bronchioles in Swiss albino mice (15 ppm, 4h)^1^	Hyperplasia of respiratory epithelium and “chronic inflammation” in male and female B6C3F1 mice (10 ppm, 2 yrs)^2^	Bronchiolar adenomas in female B6C3F1 mice only (30 ppm, 2yrs)^2^	No effects in bronchioles in male Swiss Webster rats (NOAEL: 100 ppm, 4h)^3^	No lesions in bronchioles in male and female F344/N rats (NOAEL: 60 ppm, 2yrs)^4^	No lung tumors in F344/N rats (NOAEL: 60 ppm, 2yrs)^4^	Concordant with the MOA for female mice but not male
Ethylbenzene (100-41-4)	“No active lesions” in small airways of B6C3F1 mice (750 ppm, 5 days)^5^	Alveolar metaplasia in male B6C3F1 mice (750 ppm, 2yrs)^6^	Alveolar/bronchiolar adenoma in male B6C3F1 mice (750 ppm, 2yr)^6^	No data	No lesions in Fischer rats (NOAEL: 750 ppm, 2 yrs)^6^	No lung tumors in Fischer rats (NOAEL: 750 ppm, 2 yrs)^6^	Concordant with the MOA
Coumarin (91-64-5)	Necrosis in female B6C3F1 mice (200 mg/kg, 1 day)^7^	No lesions in male or female B6C31 mice (NOAEL: 200 mg/kg, 13 wks and 2 yrs)^8^	Increased adenoma/carcinoma in male but not female B6C3F1mice (200 mg/kg, 2 yrs)^8^	No data	No lesions in F344 rats (NOAEL: 300 mg/kg, 13 wks)^9^	No lung tumors in F344 rats (NOAEL: 300 mg/kg, 13 wks, NTP 2003)	Concordant for the adverse outcomes, but not key events, no hyperplasia was present in mice
Cumene (98-82-8)	No data	Hyperplasia and metaplasia in male and female B6C3F1 mice (250 ppm, 2 yrs)^10^	Increased adenoma and carcinoma in male and female B6C3F1 mice (250 ppm, 2 yrs)^10^	No data	No lesions in F344/N rats (NOAEL: 1000 ppm, 2 yrs)^10^	No lung tumors in F344/N rats (NOAEL: 1000 ppm, 2 yrs)^10^	Concordant with the MOA
α-methylstyrene (98-83-9)	No data	No lesions in B6C3F1 mice (NOAEL: 600 ppm, 13wks and 2 yrs)^11^	No lung tumors in B6C3F1 mice (NOAEL: 600 ppm, 2 yrs)^11^	No data	No lesions in F344/N rats (NOAEL: 1000 ppm, 13 wks and 2 yrs)^11^	No lung tumors in F344/N rats (NOAEL: 1000 ppm, 2 yrs)^11^	Not concordant based on chemical analogy; possibly a function of potency
Benzofuran (271-89-6)	No data	Hyperplasia in male and female B6C3F1 mice (60 mg/kg, 2yrs)^12^	Increased alveolar/bronchiolar adenoma/carcinoma in male and female B6C3F1 mice (120 mg/kg, 2yrs)^12^	No data	No lesions in F344/N rats (NOAEL: 120 mg/kg, 2 yrs)^12^	No lung tumors in F344/N rats (NOAEL: 120 mg/kg, 2 yrs)^12^	Concordant with the MOA

All inhalation exposures summarized in this table were administered 5 days/week for the specified duration.

1 Phimister et al. 2004;

2 NTP 1993;

3 West et al. 2001;

4 NTP 2000;

5 Stott et al. 2003;

6 Chan et al. 1998;

7 Born et al. 1998;

8 NTP 1993;

9 NTP 1993;

10 NTP 2009;

11 NTP 2007;

12 NTP 1989.

**Table 7.** Weight-of-evidence summary of Evolved Bradford-Hill considerations for the hypothesized Mode of Action.

Evolved Bradford-Hill Considerations	Direct Evidence	Indirect Evidence	Contradictory Evidence	Missing Evidence
1. Biological Concordance Is the evidence for the proposed MOA consistent with broader biological knowledge?	Bronchiolar hyperplasia is well-established as a preneoplastic lesion in rodents^1^	Hypothesis that protein adduct formation and/or GSH depletion leads to bronchiolar cell death^2^	None (i.e., MOA does not directly violate broader biological knowledge)	Unknown mechanistic relationship between bronchiolar cell death and hyperplastic response
2. Essentiality of Key Events Does the evidence demonstrate that earlier key events are essential for the outcome?	Bronchiolar cell death and hyperplasia are abrogated in Cyp2F2-null mutants^3^	Direct evidence of Cyp2F2 essentiality for all intermediate KEs in tumor-resistant C57Bl/6 mice suggests that Cyp2F2 is essential for tumor responses in susceptible strains	No direct counterfactual evidence (i.e., bronchiolar tumors following specific inhibition/knockout of Cyp2F2) was identified	Direct evidence of essentiality of Cyp2F2 in bronchiolar tumors is not available with current research tools
3. Concordance of Empirical Observations Are the patterns of dose-response, temporality, and incidence for styrene induced tumours and KEs consistent with the hypothesized MOA?	Patterns are as expected in well-controlled studies in mice (see Tables 4 and 5)	No indirect supporting evidence	No evidence contradicting expected patterns	No evidence missing
4. Consistency Are results from different test systems consistent with the understanding of the hypothesized MOA?	Neither KEs nor the AO are observed in species or mutant strains that do not express Cyp2F2 and are only observed in test systems that have functional Cyp2F2	No indirect supporting evidence	Susceptibility to bronchiolar tumors is variable across mouse strains despite similar expression of Cyp2F2^4^	Impact(s) of interaction between Cyp2F2-mediated styrene toxicity and susceptible tumor backgrounds in mice is unknown
5. Analogy Do similar chemicals cause similar effects in relevant test systems consistent with the hypothesized KERs?	Data for four out of six Cyp2F2-metabolized analogues are fully concordant with the hypothesized MOA in mouse and rat test systems (see Table 6). Data for napthalene is concordant for female mice only	No indirect supporting evidence	Coumarin caused bronchiolar tumors in male B6C3F1 mice, but did not cause interim hyperplasia of bronchioles. Napthalene did not produce the AO in male mice	Available study data for α-methylstyrene likely does not use sufficiently high doses

1 Renne 2009;

2 Hill and Conolly 2019;

3 Cruzan et al. 2017;

4 Andersen et al. 2017.

**Table 8.** Species concordance of proposed key events between the mouse and human.

Key Events as Hypothesized in the Mouse	Qualitative Concordance to Human	Quantitative Concordance to Human	Evidence Base and Confidence
KE1: Conversion of Styrene to One or More Cytotoxic Metabolite(s)	Uncertain whether human enzymes produce the toxic metabolite, but qualitatively possible^1^	Human respiratory tract tissues metabolize styrene to oxide at orders of magnitude lower rate than mouse^2,3^	No direct counterfactual evidence, but general metabolic differences speak strongly against human relevance
KE2: Airway Cell Death Following Xenobiotic Metabolism	Bronchiolar club cells are frequent targets of chemical toxicity in mice but not humans due to the different levels of metabolic activity in terminal bronchioles^4^	Quantitative human data not available in to compare susceptibility of bronchiolar cells	Evidence is consistent that mice are more susceptible to injury from xenobiotic metabolism
KE3: Hyperplasia of Airway Epithelium Following Cell Death	Possible site and tissue concordance between mouse and human, but the relationship to toxic injury is unknown in human^4,5,6^	Quantitative human data not available to compare rate and sensitivity of the response	Hyperplasia of airways is a common chemically induced lesion in mice, but is not known to be in humans^4^
AO: Increased Incidence of Lung Tumors and Cancer	Bronchiolar adenoma/carcinoma is a common background tumor in mice and bears some tissue and site concordance to peripheral lung tumors in humans^5,7^	Available epidemiological data does not suggest increase of any human lung cancers associated with styrene exposure^8,9^	Evidence supports some biological concordance of peripheral lung tumors, but the significance of this is unknown

1 Vodicka et al. 2006;

2 Csanády et al. 2003

3 Sarangapani et al. (2002);

4 Pandiri 2015;

5 Dixon et al. 2008;

6 Mori et al. 2001;

7 Nicholson et al. 2022;

8 Bertke et al. 2018;

9 Daniels and Bertke, 2020.

## Appendix A: Summaries of studies exposing rats or mice to styrene

The National Cancer Institute (NCI 1979) exposed male and female Fisher 443 rats to 0, 500, 1000, or 2000mg/kg styrene *via* gavage 5 days/wk in groups of 50 animals/dose/sex in a 2-year study. The 500mg/kg group was dosed for 103 wks, while the two higher dose groups were dosed for 78 wks and then given 27 wks to recover. Male and female B6C3F1 mice were dosed 50 animals/sex/dose to a group with 0, 150, or 300mg/kg styrene *via* gavage for 5 days/wk for 78 wks with a 13 wk recovery. Male and female rats developed bronchiolar tumors at an incidence of 0-5% that was unaffected by styrene exposure. In male mice, bronchiolar tumors were absent in the vehicle group, but incidences of adenomas and carcinomas were 8% and 10% in the high dose group. Vehicle-treated female mice had no lung tumors, while adenomas were observed in 6% of female mice in the high dose group. The upward trend of bronchiolar tumors with increasing styrene is dose-dependent and reaches statistical significance for combined adenomas and carcinomas in male mice, but the authors could not reach a “firm conclusion of carcinogenicity” because of past observations of these tumors in control male mice.

Ponomarkov and Tomartis (1978) exposed groups of pregnant BD IV rats, O20 mice, and C57Bl/6 mice to styrene or vehicle by gavage on day 17 of gestation. Rats received 500mg/kg/wk, O20 mice received 1,350mg/kg/wk, and C57Bl/6 received 300mg/kg/wk. Male and female offspring then received weekly gavages at the same doses for 2 years. Rats showed little or no evidence of lung tumors in any group. Male and female O20 offspring treated with styrene experienced poor survival over the 2-yr exposure period. Background incidences for lung adenomas/carcinomas combined in O20 mice were 53% and 64% in females and males, respectively, and 100% and 89% in treated groups. Male and female C57Bl/6 offspring exposed to styrene showed low incidences of lung tumors at a rate of 4% for both sexes. Control C57Bl/6 incidences ranged from 2-25% across males and females. Untreated and vehicle-treated male mice had higher background incidences of lung tumors than females, but the tumors were not further described nor was there a significant difference from styrene-treated.

Cruzan et al. (1998) exposed male and female CD-1 (Sprague-Dawley) rats to styrene *via* inhalation at concentrations of 0, 50, 200, 500, or 1000ppm for 104 wks with 70 animals/dose/sex per group. Lung tumors were absent in all groups with the exception of one animal in the 200ppm group. No adverse non-cancer lung effects were noted in any group.

Cruzan et al. (2001) exposed male and female CD-1 (Swiss albino) mice in groups of 50 mice/sex/dose to 0, 20, 40, 80, or 160ppm for 5d/wk for up to 104 wks. Histopathological examination at 12, 18, and 24months showed bronchiolar epithelial hyperplasia in terminal bronchioles and alveolar ducts. The incidence of these lesions increased with dose and time across all groups. The background incidence of hyperplasia at 24months was 18/50 in male mice and 6/50 in female mice. Lung adenomas occurred at a background incidence of 15/50 in males and this was significantly increased to 35/50 at 40ppm styrene. In females, the background incidence of adenomas was 6/50, and a significant increase to 16/50 was seen at 20ppm. In both sexes, the increase in bronchiolar tumors appeared to plateau between 20 and 40ppm. The styrene-induced tumor increases were not seen in interim 12- and 18-month timepoints and were only observed at the end of the study.

Cruzan et al. (2017) exposed male CD-1 and C57Bl/6 mice to 120ppm styrene for 5d/wk for 104 wks. Another treatment group used Cyp2F2-knockout (KO) mice on a C57Bl/6 background, and a further group used C57Bl/6 mice knocked out for Cyp2F2 but expressing a human Cyp2F1 transgene (TG). All groups used 70 animals/group. Groups of 10 animals were used for interim timepoints of 1, 26, 52, and 78 wks, and cell proliferation in bronchioles at these interim times was measured using nuclear staining. Exposure to styrene resulted in bronchiolar hyperplasia in both CD-1 and wild-type C57Bl/6 at the end of the study and at all interim timepoints. Degeneration of terminal bronchiole cells was evident at 1 and 26 weeks in both strains, and cell proliferation was elevated at week 1 in both strains. The KO and TG groups showed no hyperplasia, degeneration, or increase in proliferation at any timepoint. At the end of the study, the incidence of bronchioloalveolar carcinomas was increased from 7/67 at background to 17/67 in styrene-exposed CD-1 mice. The incidence of bronchioloalveolar adenomas was 15/67 at background and did not increase with styrene treatment. Some discrepancies between the incidences of adenomas compared to carcinomas observed in the 2001 study using CD-1 mice are noted; these may be because of inconsistencies in classification of these lesions between the different teams providing pathology support for these studies. In C57Bl/6 mice, few or no lung tumors were seen in vehicle- or styrene-treated mice, regardless of whether WT, KO, or TG C57Bl/6 mice were being tested.

Andersen et al. (2017) exposed male C57Bl/6 mice to 0, 10, 40, and 120ppm styrene for 6h with 10 animals per group. Also tested were Cyp2F2 KO mice and Cyp2F1-humanized TG mice. Lungs were examined 24h after exposure. Additionally, active proliferation was measured with Ki-67 nuclear staining and global gene expression analysis was analyzed. Examination of the lungs showed “minimal to mild degeneration, necrosis, exfoliation, and/or attenuation of bronchiolar epithelium” in 5/10, 8/10, and 8/10 mice exposed to 10, 40, and 120ppm, respectively. The Ki-67 labeling index was marginally increased in what appeared to be a dose-responsive trend. No effects of any kind were seen in KO or TG mice. Gene expression analysis of lung tissue in wild-type C57Bl/6 showed upregulation of pathways associated with metabolism, cytokine signaling, DNA synthesis and repair, and cell cycle.

## Abbreviation List:

AO: adverse outcome
BMD: benchmark dose
CCSP: club cell secretory protein (CC10)
GSH: glutathione
KE: key event
KER: key event relationship
MOA: mode of action
NOAEL: no-observed-adverse-effect-level
NTP: National Toxicology Program
ppm: parts per million
WOE: weight of evidence
